# Characteristics, Risk Factors, and Treatment Practices of Known Adult Hypertensive Patients in Saudi Arabia

**DOI:** 10.4061/2010/168739

**Published:** 2011-01-20

**Authors:** N. Al-Hamdan, A. Saeed, A. Kutbi, A. J. Choudhry, R. Nooh

**Affiliations:** ^1^Department of Community Medicine, Faculty of Medicine-King Fahad Medical City, King Saud Bin Abdulaziz University for Health Sciences, Riyadh, Saudi Arabia; ^2^Non-Communicable Disease Department, Ministry of Health, Riyadh, Saudi Arabia; ^3^Field Epidemiology Training Program, Ministry of Health, Riyadh, Saudi Arabia

## Abstract

*Objective*. To determine the prevalence, risk factors, characteristics, and treatment practices of known adult hypertensives in Saudi Arabia. *Methods*. Cross-sectional community-based study using the WHO stepwise approach. Saudi adults were randomly chosen from Primary Health Care Centers catchment areas. Data was collected using a questionnaire which included sociodemographic data, history of hypertension, risk factors, treatment practices, biochemical and anthropometric measurements. Collected data was cheeked, computer fed, and analysed using SPSS V17. *Results*. Out of 4719 subjects (99.2% response), 542 (11.5%) subjects were known hypertensives or detected by health workers in the past 12 months. Hypertension was significantly associated with age, gender, geographical location, education, employment, diabetes, physical inactivity, excess body weight, and ever smoking. Multiple logistic analysis controlling for age showed that significant predictors of hypertension were diabetes mellitus, ever smoking, obesity, and hypercholesteremia. Several treatment modalities and practices were significantly associated with gender, age, education, and occupation. About 74% were under prescribed treatment by physicians, 62% on dietary modification, 37% attempted weight reduction, 27% performed physical exercise, and less than 7% used herbs, consulted traditional healers or quitted smoking. Income was not significantly associated with any treatment modality or patient practices. *Conclusion*. Hypertension (known and undetected) is a major chronic health problem among adults in Saudi Arabia. Many patients' practices need changes. A comprehensive approach is needed to prevent, early detect, and control the disease targeting, the risk factors, and predictors identified.

## 1. Introduction

The burden of noncommunicable Diseases (NCD) is rising rapidly nationally and globally constituting a major challenge to development. The World Health Organization (WHO) in recognition of this trend developed a global strategy for the prevention and control of noncommunicable diseases [1]. This strategy focuses on assessing the pattern and trends of risk factors of major NCD; the national capacity for prevention and control; promoting the development of evidence-based strategy to reduce unhealthy behaviors and major risk factors; implementing cost-effective and equitable interventions for the management of common noncommunicable diseases [1]. The Kingdom of Saudi Arabia (KSA) is considered to be one of the rapidly growing countries that have been affected by the lifestyle changes reflected by change in disease pattern. Data on NCD and their risk factors was either very scanty or not collated in the country [2]. Therefore, it was decided to carry out a national situational analysis and a baseline survey of NCD risk factors. Hypertension was one of the major diseases covered in the survey. 

Hypertension is an important worldwide public-health challenge because it is one of the most common chronic conditions. It may not be detected and if detected may be neglected by many individuals particularly in the developing world. This is unfortunately because it is a major risk factor for heart disease, stroke, kidney disease and other complications. The prevalence of hypertension continues to rise across the world, and most patients who receive medical intervention are not adequately treated to goal [3]. The reported prevalence of hypertension varied around the world from 3.4% in men in rural India as the lowest to 72.5% in elderly women in Poland as the highest [4]. Earlier community surveys in KSA showed that the overall prevalence among adults in the country ranged from 15.4 to 26.1% depending on the hypertension definition used [5, 6]. Accurate updated data of the countrywide prevalence of known and undetected cases of this condition is essential. This primary information is vital for rational planning of preventive and curative health services. This communication focuses on the characteristics, risk factors and practices of adult hypertensives. Those are adult Saudi subjects who are known or were informed by health workers to be hypertensives during their usual visits to the health facilities in the past 12 months.

## 2. Methods

This is a cross-sectional community-based study covering whole of Kingdom of Saudi Arabia. The WHO stepwise approach to Surveillance (STEPS) of NCD risk factors was the basis for conducting the survey and collecting data [7].

**Study Population**

The study population was all Saudi population of all the 20 health regions of the country aged 15–64 years.

**Sampling**

A multistage stratified cluster random sampling technique was used to recruit the study subjects. Stratification was based on age (five 10-year age groups), gender (2 groups), and health regions of country. Based upon proposed methodology of the WHO stepwise approach, a sample size of 196 was calculated for each of these ten strata. A list of all Primary Health Care Centers (PHCCs) in each region was prepared, and 10% of these PHCCs were randomly chosen, and allocated regional sample to them proportionate to the size of their catchment population in sampled PHCCs. To identify the households, a map of the health center coverage area was used to choose the houses. Each house was assigned a number and a simple random draw was made. The total selected sample included 4758 adult subjects with females constituting 50.8% of them. Females constituted 38.2% in the age groups 15–44 years compared to 32.7% of males. For age groups 45 years and above, males constituted 16.5% compared to 12.6% for females.

### 2.1. Data Collection

#### 2.1.1. Tool Used

Data was collected using the WHO stepwise approach which includes a questionnaire, physical measurements and biochemical measurements covering hypertension and other chronic diseases and risk factors. The questionnaire was translated into Arabic by a team of physicians and was back translated to ensure the accuracy of translation. Arabic instrument was pretested on 51 eligible respondents for wording and understanding of the questions, and necessary adjustments were made in the instrument in light of the pretest. The questionnaire includes socio-demographic data, history of blood pressure and blood pressure measurement in addition to other chronic diseases and risk factors. A specific question enquired whether the subject is a known hypertensive or discovered to be hypertensives by health professionals during the last 12 months.

#### 2.1.2. Data Collectors

Data was collected by 54 males and 54 female collectors who work in teams. Each field team was made up of four persons; a male data collector, a female data collector, a driver, and a female assistant. Data collection teams were supervised by a hierarchy of local supervisor, regional coordinators, and national coordinator.

### 2.2. Training of Data Collectors

All individuals involved in data collection attended a comprehensive training workshop that included interview techniques, data collection tools, practical applications and field guidelines.

### 2.3. Blood Pressure Measurement

Blood pressure (BP) measurements were taken using a digital sphygmomanometer. Before taking the measurements, the respondent was advised to sit quietly and rest for 5 min with the legs uncrossed and the right arm free of clothing. Then, the right arm was placed on the table with the palm facing upwards. The appropriate cuff size was selected. Three measurements were recorded with five-minutes intervals. The average of the three readings was considered the actual blood pressure. The study sample for the present communication was all known adult hypertensives and all those informed by health professionals to be hypertensives during the past 12 months during their usual visit to health facilities. They do not include newly diagnosed hypertensives during the survey itself.

### 2.4. Data Management and Analysis

Questionnaires collected from the field were reviewed by team leaders assigned to each team before submitting them to the headquarters for data entry. Double entry of the questionnaires was performed using EPI-INFO 2000 software and EpiData software developed by the Menzes Centre for validation. After data entry, data cleaning was conducted. New variables were defined by adopting the standard steps variables (STEPS Data Management Manual, Draft version v1.5, October 2003).

### 2.5. Statistical Analysis

Data analysis was conducted using Statistical Package for Social Sciences (SPSS) software. Univariate analysis was performed for significant associations and Logistic Regression analysis was used for significant predictors of hypertension. A *P* value of ≤.05 was taken for statistical significance. The data were processed using SPSS version 17.

### 2.6. Ethical Clearance and Confidentiality

The protocol and the instrument of the surveillance were approved by the Ministry of Health, Center of Biomedical Ethics and the concerned authorities in the Kingdom. Informed consent of all subjects was obtained. Confidentiality of data was assured and that data will be used only for the stated purpose of the survey.

## 3. Results

**Figure 1:**
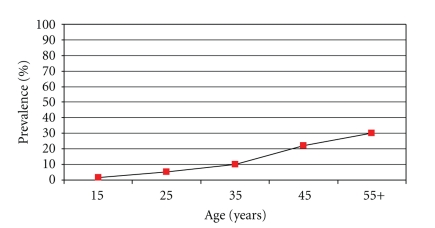
Prevalence of hypertension (%) by age in Saudi Arabia 2005.

**Figure 2:**
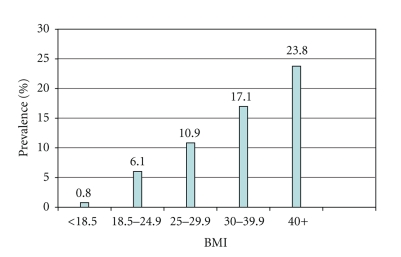
Prevalence in percentage of hypertension by BMI in Saudi Arabia, 2005.

A total of 4758 subjects participated in the study and 4719 were included in the final analysis giving an overall of 99.2% response rate. Females constituted about 51% of the study population. Females in age group 25–44 years were more than males (53.3% compared to 43.1%), while males were more than females in the age group 45–64 years (33.5% compared to 24.8%). Of the total participating subjects, 542 were known or detected hypertensives during the past 12 months by health professionals giving an overall prevalence of 11.5%. Hypertension prevalence increases with increasing age and BMI as shown in Figures 1 and 2. Univariate analysis showed that there were significant genderand age differences. Females had significantly higher prevalence than males (12.8% compared to 10.2%). Hypertension significantly increased with advancing age in both genders. There were also significant regional differences. The highest prevalence was in the central region and the lowest was in the southern region (15.3% compared to 7.6%) as can be seen in Table 1. Prevalence of hypertension was also significantly higher in illiterates and postuniversity educated, retired, and unemployed unable subjects. It was lowest among students. The association of hypertension prevalence with income was studied. Subjects with very low or very high family income tended to have higher hypertension prevalence, but the differences were not statistically significant. Hypertension was significantly higher in subjects who were diabetic, hypercholestremic, ever smoking, physically inactive with higher Body Mass Index (BMI), and central obesity as depicted in Table 2. All significant factors studied in univariate analysis were studied for significant hypertension predictors using multiple logistic regression analysis after controlling for age. Obesity, ever smoking, diabetes mellitus, and hypercholesteremia were significant predictors of hypertension as can be seen in Table 3. Patients' treatment modalities and practices are depicted in Table 4. About 74% of patients were on prescribed treatment in form of medications by physicians, and less than 7% were getting advice from traditional healers or taking herbal medications. There was significant association between practices and demographic characteristics of patients. Females used prescribed treatment, dietary control, and herbs significantly more than males, while males quitted smoking as a treatment modality significantly more than females. Older patients used treatment, medications, dietary control, weight reduction significantly more than younger patients. The central region patients used weight reduction and exercise significantly more than the other regions. 

Treatment modalities and prescribed treatment by physicians were significantly more associated with lower educational level, while dietary control was significantly more practiced by university graduates. Further analysis showed that retired, and unemployed patients used prescribed treatment significantly more than the other occupations. Students used all treatment modalities less than all other subjects. Income was not significantly associated with any treatment modality.

## 4. Discussion

The overall prevalence of known adult hypertension in this study was 11.5%. Similar STEPwise surveys reported an average prevalence rate 11%-12% (ranging from 8.5 to 19.2%) [8–10]. These rates are much less than the overall rates found in this STEP wise study and the last national survey which showed that one-fifth or more of the adult population were hypertensives [2, 6]. This shows that many hypertensives are not recognized and not detected. This appears to confirm that the rule of halves in hypertension is still valid as confirmed by many studies later in many parts of the world [11, 12]. The situation is complicated by undetected hypertensives by health workers, a trend known as masked hypertension with prevalence reported to affect 10%–16.8% of the general population and carried an adverse prognosis, both in terms of increased target organ damage and cardiovascular events [13, 14] This study showed the significant relation of hypertension with advancing age in both sexes in agreement with national and international studies in almost all populations with diverse geographical, cultural, and socioeconomic characteristics [2, 5, 6, 8–10]. In this study the prevalence of hypertension among females was significantly higher than males. Similar STEPwise surveys reported that females in many Arab and Moslem countries including neighboring countries showed higher hypertension prevalence than males [8–10]. Overall worldwide prevalence of hypertension, however, showed no significant gender difference [4, 15]. These results show that gender has no consistent association with hypertension, and differences may be due to confounding variables. This study found significant association between hypertension and occupation and education status. Illiterate and highly educated, unemployed and subjects doing house duties tended to have significantly higher hypertension prevalence. In neighboring Oman, hypertension prevalence was higher among subjects with lower educational level, retired and housewives [9]. Occupation was significantly associated with hypertension in some Asian countries. Farmers, as an example, in Vietnam suffer from hypertension less than traders and handicraft makers [16]. Hypertension was more in Japan in jobs characterized by shift work, awkward posture, exposure heat, sitting work, doing several tasks simultaneous, being interrupted at work not being able to take eyes off work [17]. Other countries outside Asia such as Germany showed gender differences in the association of hypertension with occupation. In men the highest prevalence of hypertension was in metal-processing workers, carpenters/painters, and electricians, compared to office clerks. In women, the highest prevalence was found in technicians/forewomen, scrutinizers/storekeepers, and food-processing occupations [18]. These differences may be due to a specific occupational hazard or may be due to several confounders associated with hypertension such as education and income which may be closely related to occupation. This study showed that hypertension tend to be more among low- and high-income subjects although results did not reach significance level. Some studies reported that in both men and women, the income distributions of blood pressure and hypertension were nonlinear, indicating elevated levels in low as well as in high-income groups [19]. Low and high incomes may be associated with psychological tensions which may be associated with hypertension. Education; occupation, and income are all related to socioeconomic status (SES). Low SES is associated with elevated rates of blood pressure-related cardiovascular disease [20, 21]. Our findings showed significant geographical variation in hypertension prevalence where the Central Region had the highest and the southern region had the lowest prevalence of hypertension. The Central Region is highly urbanized, industrialized, and developed compared to the southern region. Geographical variations in the prevalence of hypertension were reported in many studies in different regions of the world [9, 22–26]. These regional variations in blood pressure may also be related to regional variation in socioeconomic, demographic and dietary in addition to the geographic characteristics. Significant hypertension predictors as revealed by multivariate logistic regression analysis included obesity, ever tobacco smoking, diabetes mellitus and high cholesterol level. This is in agreement with many, national and international studies which showed that hypertension was threefold more in diabetics, significantly more in obese and ever smokers and those with high levels of total cholesterol [27–31]. The situation is very worrying when we know that the prevalence of obesity, diabetes, dyslipidemia and smoking among adults in KSA has reached alarming magnitudes affecting more than a quarter of the population [32–34].

The management of hypertension entails using pharmacological, nonpharmacological or both interventions. The practices of patients to control their disease status in this study include dietary modification (61.6%), weight reduction (37%) and exercise (27.1%). Such modalities and others nonpharmacological interventions were found to be very useful in lowering blood pressure. Lifestyle interventions can have a similar reduction in blood pressure to single antihypertensive drugs [34]. Weight loss, especially when combined with dietary sodium restriction, fruits and vegetables, exercise, tobacco-free environment lower blood pressure in hypertensive and also in normotensive patients [35–39]. It is emphasized that simple advice from physicians can have a positive influence on patients' motivation to make lifestyle changes [32, 34]. The suitable premises for physical activity should be provided taking in consideration religious and cultural aspects. It is gratifying that only few patients were using herbs or consulting traditional healers for treatment of hypertension. Many of these were not proved to be effective or safe, and such practices should be strongly discouraged.

## 5. Conclusion

Hypertension is an important chronic health problem among adults in KSA. It is significantly associated with advancing age, excess body weight, smoking, dyslipidemia, diabetes mellitus, lower level of education, and employment status. Undetected hypertension is a major problem. A comprehensive approach is needed to prevent, early detect and control the disease aiming at preventing, reversing or reducing the risk factors. This should start as early as possible among school students. Emphasis should be on the importance of healthful lifestyle behaviors. All health care providers particularly in PHCCs should check blood pressure for all clients properly and repeatedly, enquire about risk factors, and offer advice concerning lifestyle modifications particularly encouraging regular physical activity, proper nutritional practices and avoiding smoking. Public health policies should be enforced to provide a favorable environment for hypertension control by regulating food industry, providing facilities for physical activity and smoking cessation services.

**What Is Already Known of This Topic**

Hypertension is a common chronic morbidity increasing worldwide including KSA. The magnitude of the problem is not accurately assessed.

**What This Study Adds**

This study confirms that detected hypertension is a real problem in Saudi Arabia. Many practices of patients are significantly associated with their demographic characteristics. Undetected hypertension is of equal magnitude and importance. Health professionals have pivotal role in detecting new cases by regularly enquiring about the risk factors and measuring blood pressure correctly during each client visit. Intervention strategies need to address the identified predictors and significant risk factors.

## Figures and Tables

**Table 1:** Prevalence of hypertension according to the sociodemographic characteristics.

Variable	Total number	Hypertensives (*N* = 542)% (*n*)	*P* value
Gender	(*n* − 4719)		
Male	2323	10.2 (236)	.005
Female	2396	12.8 (306)	

Age (years)	(*n* = 4719)		<.001
15–24	1058	1.8 (19)	
25–34	1121	5.1 (57)	
35–44	1163	10.1 (117)	
45–54	836	22.1 (185)	
55–64	541	30.3 (164)	

Region	(*n* = 4719)		
Central	1112	15.3 (170)	<.001
Eastern	706	11.9 (84)	
Northern	452	10.6 (48)	
Southern	998	7.6 (76)	
Western	1451	11.3 (164)	

Work	(*n* = 4713)		
Government	1371	9.1 (124)	<.001
Non government	179	10.7 (19)	
Self employed	275	12.9 (35)	
Students	649	1.3 (8)	
House-maker	1759	15.4 (269)	
Retired	308	23.8 (73)	
Unemployed (able)	181	3.4 (6)	
Unemployed (not able)	29	26.7 (8)	

**Table 2:** Prevalence of hypertension according to level of physical activity, obesity, smoking, diabetes, and cholesterol level.

Variable	Total subjects	Hypertensive subjects	*P* value
Physical activity	(*n* = 4576)		.001
High >120 minutes/day	762	7.3 (56)	
Moderate 30–120 minutes/day	766	9.9 (76)	
Low <30 minutes/day	3048	12.0 (366)	

BMI	(*n* = 4560)		<.001
(weight in kilograms/height in meters squared)			
<18.5	256	0.8 (2)	
18.5–24.9	1188	6.1 (72)	
25.0–29.9	1467	10.9 (160)	
30.0–39.9	1414	17.1 (242)	
40.0+	235	23.8 (56)	

Ever smoking	(*n* = 4576)		.023
(Tobacco use in the past irrespective of quantity or duration)			
Yes	580	14.5 (84)	
No	3996	11.2 (449)	

Diabetes	(*n* = 4639)		<.001
(Known diabetic or fasting blood glucose ≥7.00 mm/Liter)			
Yes	709	32.2 (228)	
No	3930	7.8 (307)	

Central obesity: males (Waist W/H ratio >1.0) Females (W/H ratio >0.85)	(*n* = 4385)		<.001
Yes	963	17.5 (169)	
No	3422	356 (10.4)	

Total cholesterol level	(*n* = 4469)		.001
Elevated >5.2 mmol/Liter	862	17.1 (147)	
Not elevated ≤5.2 mmol/Liter	3607	10.4 (374)	

**Table 3:** Logistic regression analysis of hypertension contributing factors.

Items	B	S.E.	Wald X2 value	DF	*P* value	Odds ratio (OR)	95% C.I. for OR	
							Lower	Upper
Ever Smoking	1.141	0.417	7.496	1	.006	3.129	1.383	7.079
Diabetes mellitus	1.681	0.436	14.883	1	<.001	5.373	2.287	12.623
Central obesity	1.353	0.477	8.034	1	.005	3.870	1.518	9.865
Total cholesterol (mmol/Liter)	0.276	0.123	5.028	1	.025	1.317	1.035	1.676
Constant	6.755	1.952	11.970	1	.001	0.001		

Covariates in equation: gender, region, occupation, education, smoking, diabetes, central obesity, physical activity, cholesterol level.

**Table 4:** Treatment modalities and practices of hypertensives (*n* = 542).

Variable	Any treatment (%)	Prescribed (%)	Diet (%)	Weight loss (%)	Stop smoking (%)	Exercise (%)	Traditional (%)	Herbs (%)
Gender								
Male	86.4	75.4	53.0	35.2	13.6	31.4	5.1	2.1
Female	91.9	71.2	68.3	38.6	1.3	23.9	7.8	6.2
*P* value	.043	.277	<.001	.418	<.001	.052	.201	.022

Age (Years)								
15–24	52.6	26.3	31.6	21.1	15.8	21.1	0.0	0.0
25–34	71.9	35.1	45.6	28.1	7.0	21.1	8.8	7.0
35–44	87.2	62.4	65.8	48.7	3.4	31.6	4.3	3.4
45–54	93.0	81.1	62.2	36.8	7.6	26.5	7.0	4.3
55–64	97.7	90.2	67.1	34.1	6.7	27.4	7.9	4.9
*P* value	<.001	<.001	.003	.002	.309	.616	.568	.709

Region								
Central	90.0	72.4	67.1	48.8	4.1	25.3	3.5	2.4
Eastern	90.5	70.2	58.3	36.9	6.0	31.0	6.0	3.6
North	98.6	79.2	43.8	20.8	8.3	20.8	8.3	4.2
South	90.8	71.1	65.8	32.9	17.1	35.5	6.6	2.6
West	87.8	74.4	61.0	31.7	4.3	25.0	9.8	7.9
*P* value	.943	.810	.048	.001	.002	.291	.239	.125

Education								
Non	93.7	81.8	64.4	33.6	5.5	24.2	8.7	3.2
Primary	91.5	80.1	62.4	39.0	5.7	29.2	7.8	6.4
Intermediate	77.1	47.9	43.8	35.4	1.4	31.2	0.0	0.0
Secondary	68.4	42.1	47.7	36.8	7.9	26.3	10.5	7.9
University+	87.8	51.0	75.5	46.9	12.2	30.6	6.1	4.1
Vocational	100	92.3	61.5	53.8	0.0	38.3	7.7	15.4
*P* value	<.001	.001	.019	.391	.378	.699	.455	.100

Total (%)	73.1	73.7	61.6	37.1	6.6	27.1	6.6	4.4

## References

[1] WHO Prevention and control of noncommunicable diseases: implementation of the global strategy Report by the Secretariat.

[2] http://www.who.int/chp/steps/2005_SaudiArabia_STEPS_Report_EN.pdf.

[3] Bakris G, Hill M, Mancia G (2008). Achieving blood pressure goals globally: five core actions for health-care professionals. A worldwide call to action. *Journal of Human Hypertension*.

[4] Lawes CM, van der Hoorn S, Rodgers A (2008). Global burden of blood-pressure-related disease, 2001. *The Lancet*.

[5] Wahid Saeed AA, Al Shammary FJ, Khoja TA, Hashim TJ, Anokute CC, Khan SB (1996). Prevalence of hypertension and sociodemographic characteristics of adult hypertensives in Riyadh city, Saudi Arabia. *Journal of Human Hypertension*.

[6] Al-Nozha MM, Abdullah M, Arafah MR (2007). Hypertension in Saudi Arabia. *Saudi Medical Journal*.

[7] Bonita R, de Courten M, Dwyer T, Jamorzik K, Winkelmann R Surveillance of risk factors for Non Communicable diseases.

[8] http://www.who.int/chp/steps/kuwait/en/index.html.

[9] http://www.who.int/chp/steps/oman/en/index.html.

[10] http://www.who.int/chp/steps/egypt/en/index.html.

[11] Scheltens T, Bots ML, Numans ME, Grobbee DE, Hoes AW (2007). Awareness, treatment and control of hypertension: the ’rule of halves’ in an era of risk-based treatment of hypertension. *Journal of Human Hypertension*.

[12] Deepa R, Shanthirani CS, Pradeepa R, Mohan V (2003). Is the ’rule of halves’ in hypertension still valid? Evidence from the Chennai Urban Population Study. *Journal of Association of Physicians of India*.

[13] Pickering TG, Eguchi K, Kario K (2007). Masked hypertension: a review. *Hypertension Research*.

[14] Verberk WJ, Kessels AGH, de Leeuw PW (2008). Prevalence, causes, and consequences of masked hypertension: a meta-analysis. *American Journal of Hypertension*.

[15] Kearney PM, Whelton M, Reynolds K, Muntner P, Whelton PK, He J (2005). Global burden of hypertension: analysis of worldwide data. *The Lancet*.

[16] van Minh H, Byass P, Chuc NTK, Wall S (2006). Gender differences in prevalence and socioeconomic determinants of hypertension: findings from the WHO STEPs survey in a rural community of Vietnam. *Journal of Human Hypertension*.

[17] Tsutsumi A, Kayaba K, Tsutsumi K, Igarashi M (2001). Association between job strain and prevalence of hypertension: a cross sectional analysis in a Japanese working population with a wide range of occupations: the Jichi Medical School cohort study. *Occupational and Environmental Medicine*.

[18] Schumann B, Seidler A, Kluttig A, Werdan K, Haerting J, Greiser KH Association of occupation with prevalent hypertension in an elderly East German population: an exploratory cross-sectional analysis.

[19] Mendez MA, Cooper R, Wilks R, Luke A, Forrester T (2003). Income, education, and blood pressure in adults in Jamaica, a middle-income developing country. *International Journal of Epidemiology*.

[20] Forman JP, Stampfer MJ, Curhan GC (2009). Diet and lifestyle risk factors associated with incident hypertension in women. *Journal of the American Medical Association*.

[21] Grotto I, Huerta M, Sharabi Y (2008). Hypertension and socioeconomic status. *Current Opinion in Cardiology*.

[22] Rampal L, Rampal S, Azhar MZ, Rahman AR (2008). Prevalence, awareness, treatment and control of hypertension in Malaysia: a national study of 16,440 subjects. *Public Health*.

[23] Hajjar I, Kotchen T (2003). Regional variations of blood pressure in the United States are associated with regional variations in dietary intakes: the NHANES-III data. *Journal of Nutrition*.

[24] Kershaw KN, Diez Roux AV, Carnethon M (2010). Geographic variation in hypertension prevalence among blacks and whites: the multi-ethnic study of atherosclerosis. *American Journal of Hypertension*.

[25] van der Sande MAB, Milligan PJM, Walraven GEL (2001). Geographical variation in prevalence of hypertension within The Gambia. *Journal of Human Hypertension*.

[26] Reynolds K, Gu D, Muntner P (2003). Geographic variations in the prevalence, awareness, treatment and control of hypertension in China. *Journal of Hypertension*.

[27] Salman RA, Al-Rubeaan KA (2009). Incidence and risk factors of hypertension among Saudi type 2 diabetes adult patients: an 11-year prospective randomized study. *Journal of Diabetes and Its Complications*.

[28] Longo GZ, Das Neves J, Luciano VM, Peres MA (2009). Prevalence of high blood pressure levels and associated factors among adults in Southern Brazil. *Arquivos Brasileiros de Cardiologia*.

[29] Thuy AB, Blizzard L, Schmidt MD, Luc PH, Granger RH, Dwyer T (2010). The association between smoking and hypertension in a population-based sample of Vietnamese men. *Journal of Hypertension*.

[30] Masala G, Bendinelli B, Versari D (2008). Anthropometric and dietary determinants of blood pressure in over 7000 Mediterranean women: the European Prospective Investigation into Cancer and Nutrition-Florence cohort. *Journal of Hypertension*.

[31] Petrella RJ, Merikle E (2008). A retrospective analysis of the prevalence and treatment of hypertension and dyslipidemia in Southwestern Ontario, Canada. *Clinical Therapeutics*.

[32] Al-Nozha MM, Al-Mazrou YY, Al-Maatouq MA (2005). Obesity in Saudi Arabia. *Saudi Medical Journal*.

[33] Al-Nozha MM, Al-Maatouq MA, Al-Mazrou YY (2004). Diabetes mellitus in Saudi Arabia. *Saudi Medical Journal*.

[34] Bassiony MM (2009). Smoking in Saudi Arabia. *Saudi Medical Journal*.

[35] Campbell NRC, Khan NA, Hill MD (2009). 2009 Canadian Hypertension Education Program recommendations: the scientific summary—an annual update. *Canadian Journal of Cardiology*.

[36] Bhatt S, Luqman-Arafath T, Guleria R (2007). Non-pharmacological management of hypertension. *Indian Journal of Medical Sciences*.

[37] Neutel CI, Campbell NRC (2008). Changes in lifestyle after hypertension diagnosis in Canada. *Canadian Journal of Cardiology*.

[38] Wolf-Maier K, Cooper RS, Banegas JR (2003). Hypertension prevalence and blood pressure levels in 6 European countries, Canada, and the United States. *Journal of the American Medical Association*.

[39] Whelton SP, Chin A, Xin X, He J (2002). Effect of aerobic exercise on blood pressure: a meta-analysis of randomized, controlled trials. *Annals of Internal Medicine*.

